# Preanalytical Stability of CSF Total and Oligomeric Alpha-Synuclein

**DOI:** 10.3389/fnagi.2021.638718

**Published:** 2021-03-03

**Authors:** Ilham Y. Abdi, Nour K. Majbour, Eline A. J. Willemse, Wilma D. J. van de Berg, Brit Mollenhauer, Charlotte E. Teunissen, Omar M. El-Agnaf

**Affiliations:** ^1^Neurological Disorders Research Centre, Qatar Biomedical Research Institute (QBRI), Hamad Bin Khalifa University (HBKU), Doha, Qatar; ^2^Neurochemistry Laboratory, Department of Clinical Chemistry, Amsterdam Neuroscience, Amsterdam University Medical Center, Amsterdam, Netherlands; ^3^Section Clinical Neuroanatomy and Biobanking, Department of Anatomy and Neurosciences, Amsterdam Neuroscience, Amsterdam University Medical Center, Vrije University Amsterdam, Amsterdam, Netherlands; ^4^Paracelsus-Elena-Klinik, Klinikstraße, Kassel, Germany; ^5^Department of Neurology, University Medical Center Göttingen, Göttingen, Germany

**Keywords:** preanalytical factors, alpha-synuclein, oligomers, cerebrospinal fluid, biomarkers

## Abstract

**Background**: The role of cerebrospinal fluid (CSF) alpha-synuclein as a potential biomarker has been challenged mainly due to variable preanalytical measures between laboratories. To evaluate the impact of the preanalytical factors contributing to such variability, the different subforms of alpha-synuclein need to be studied individually.

**Method**: We investigated the effect of exposing CSF samples to several preanalytical sources of variability: (1) different polypropylene (PP) storage tubes; (2) use of non-ionic detergents; (3) multiple tube transfers; (4) multiple freeze-thaw cycles; and (5) delayed storage. CSF oligomeric- and total-alpha-synuclein levels were estimated using our in-house sandwich-based enzyme-linked immunosorbent assays.

**Results**: Siliconized tubes provided the optimal preservation of CSF alpha-synuclein proteins among other tested polypropylene tubes. The use of tween-20 detergent significantly improved the recovery of oligomeric-alpha-synuclein, while multiple freeze-thaw cycles significantly lowered oligomeric-alpha-synuclein in CSF. Interestingly, oligomeric-alpha-synuclein levels remained relatively stable over multiple tube transfers and upon delayed storage.

**Conclusion**: Our study showed for the first-time distinct impact of preanalytical factors on the different forms of CSF alpha-synuclein. These findings highlight the need for special considerations for the different forms of alpha-synuclein during CSF samples’ collection and processing.

## Introduction

A vast majority of studies done on neurological disorders use cerebrospinal fluid (CSF) as the biofluid of choice to explore and assess biomarkers as CSF protein levels most closely reflect the pathophysiology of the brain (Robey and Panegyres, 2019). A major subset of these neurodegenerative disorders, referred to as “synucleinopathies,” is hallmarked by the deposition of the cytoplasmic protein alpha-synuclein (α-syn) as aberrant inclusions in neurons and glial cells in the brain (Spillantini et al., 1998). Levels of different forms of α-syn in CSF have been widely investigated in several studies as potential biomarkers of synuclein aggregation disorders. Most studies that sought to explore α-syn as a biomarker focused on total α-syn (t-α-syn) levels either alone or in combination with other biomarkers with more recent studies looking at modified forms of α-syn such as oligomeric (o-α-syn) and phosphorylated α-syn at Ser 129 (pS129-α-syn) (Mollenhauer et al., 2008, 2013; Parnetti et al., 2011, 2014a,b, 2019; Majbour et al., 2016, 2020)

However, despite the growing number of studies investigating CSF α-syn biomarkers, it remains a challenge to use these biomarkers in routine diagnostic or prognostic procedures as they lack the required accuracy (sensitivity and specificity) (Wang et al., 2015; Eusebi et al., 2017). Impaired biomarker standardization has been attributed to several possible confounding factors such as inter-individual variations in CSF protein measures or clinical heterogeneity, medications, blood contamination, assays protocols, and preanalytical variations (Kang et al., 2016; Ming et al., 2016; Mollenhauer et al., 2017a,b, 2019; Willemse et al., 2019; Barkovits et al., 2020). Preanalytical factors are defined as the variables present before biomarker assessment influencing its precise evaluation (Guder, 2014). Little is yet known about the influence of these preanalytical factors on CSF α-syn measurements (Stewart et al., 2019). In the current study, we aim to understand the stability of CSF α-syn biomarkers, mainly t-α-syn and o-α-syn, against potential preanalytical factors that occur during CSF handling and processing. The novelty of this study lies in it being the first to specifically look at o-α-syn stability in CSF, which is important as growing evidence suggest that it plays an important role in the pathogenesis of synuclein aggregation disorders and may even be a better marker compared to t-α-syn (El-Agnaf et al., 2003; Park et al., 2011; Majbour et al., 2016; Williams et al., 2016; Volc et al., 2020).

## Materials and Methods

### CSF Sample Collection

Anonymous CSF samples that had inadequate clinical information or were not fit for clinical validation were collected from Alzheimer Center Biobank at the VU University Medical Center (VUmc, Amsterdam, The Netherlands) and University Medical Center (Göttingen, Germany). The samples were pooled and aliquoted as 500 μl aliquots. Samples collected from the Biobank at VUmc were used in α-syn stability and adsorption experiments while those from University Medical Center Göttingen were used in investigations done on adsorption of α-syn. Aliquots were stored at −80°C in 1.5 ml polypropylene tubes with screw caps (Sarstedt, Nümbrecht, Germany). All samples were blinded, and donors gave written informed consent at study entry for the use of clinical information and CSF material for scientific research purposes. The study was conducted according to the revised Declaration of Helsinki and Good Clinical Practice guidelines and approved by the local ethics committee of the VU University Medical Center.

### Samples Processing and Treatment

#### Adsorption to Surface Walls of Storage Tubes

To assess the extent of adsorption of α-syn to the surface walls of storage tubes, three experimental approaches were followed: (1) we tested the difference in α-syn levels when using six different polypropylene (PP) types from different vendors (see Table 1 for tube information) compared to Nunc tubes (NUNC, Rochester, NY, USA) used as control. The main samples stocks were thawed on ice and aliquoted into the different tubes and frozen back overnight at −80°C before testing the next day; (2) we looked to observe the effect of using non-ionic detergents by measuring α-syn levels in the presence and absence of 0.05% of Tween-20, Triton X-100, or NP-40 detergents. The detergent treatments were added after thawing CSF samples and then incubated at 4°C for 30 min before testing; and (3) lastly, CSF samples were exposed to up to six tube transfers before quantification with thorough mixing between transfers to measure the difference in α-syn levels following the multiple tube transfers (reference sample-no tube transfer). For all experiments except those testing different polypropylene tubes, Sarstedt 0.5 ml polypropylene tubes were used.

**Table 1:** Summary information of polypropylene tubes.

Vendor	Catalog no.	Volume (ml)	Specifications
Nunc (reference tubes)	368632	1.8	Sterile, screw cap, non-pyrogenic, cryovial
Denville scientific	C19063	0.6	Silicon-coated, sterile. Enzyme and DNA free
Corning	430791	15	Sterile, screw cap, RNase, DNase free and non-pyrogenic
Sarstedt	72.730.005	0.5	Sterile, screw cap
Eppendorf	0030.120.086	1.5	Safe-lock, sterile, free of pyrogen, RNase, DNA and ATP. Autoclavable.
Extragene	104571010	1.5	Non-sterile, flip cap, DNase and RNase and Pyrogen safe
Costar	3213	2	Snap Cap, Non-sterile

#### Alpha-Synuclein Stability-Freeze-Thaw Cycles and Delayed Storage

CSF pools were aliquoted into 19 (Sarstedt-0.5 ml) tubes per set (*n* = 3). The first five tubes of each set were subjected to 1–7 freeze-thaw cycles (reference sample-1 freeze-thaw cycle). The remaining tubes were split into two sets which were then exposed to delayed storage times: 1, 2, 4, 24, 72, or 168 h at either 4°C or room temperature (RT) before finally storing them at −80°C (the reference sample, *t* = 0, was directly stored at −80°C).

#### Oligomeric- and Total- Alpha-Synuclein Quantification

CSF total and oligomeric α-syn levels were measured using our previously described ELISA assays (Majbour et al., 2016). Briefly, 384-well Maxisorb (Nunc) microplates were coated with 0.1 μg/ml Syn-140 (sheep anti-α-syn polyclonal antibody against t-α-syn), or 0.2 μg/ml Syn-O_2_ (o-α-syn specific monoclonal antibody) at 50 μl/well in 200 mM NaHCO_3_, pH 9.6 and incubated overnight at 4°C. Plates were washed thrice in phosphate-buffered saline with 0.05% Tween-20 (PBST) then blocked with 100 μl/well of blocking buffer (PBST with 2.5% gelatin) for 2 h at 37°C. After washing plates again, CSF samples and assay standards prepared as serial dilutions of recombinant human α-syn and o-α-syn in artificial CSF were added to the plates in duplicates at 50 μl/well and incubated at 37°C for 2.5 h. Detection antibodies, 11D12 (mouse anti-α-syn monoclonal antibody) against t-α-syn and FL-140 (rabbit polyclonal antibody, Santa Cruz Biotechnology, Santa Cruz, CA, USA) against o-α-syn were used at the diluted concentration of 1:5,000 and 1:1,000, respectively. Following a washing step, 50 μl/well of species-appropriate secondary antibodies: donkey anti-mouse IgG HRP or goat anti-rabbit IgG HRP (Jackson Immuno Research, West Grove, PA, USA), was added to the respective plates at the diluted concentration of 1:20,000 and incubate at 37°C for 2 h. After a final wash, the plates were incubated with an enhanced chemiluminescent substrate (SuperSignal ELISA Femto, Pierce Biotechnology, Rockford, IL, USA; 50 μl/well). The chemiluminescence (relative light units) was immediately measured using a PerkinElmer Envision multilabel plate reader (PerkinElmer, Finland).

### Data Analysis

Standard calibrator curves for ELISA assays were fitted using the sigmoidal, 4PL, nonlinear regression model, where X = log(concentration), and CSF α-syn concentrations were calculated by interpolating from the curves. The generated figures and as well as the calculations used to establish the curves generated and determine α-syn concentrations were carried out using GraphPad Prism 7.0. The variations in the α-syn concentration due to the type of storage tube used, repeated freeze-thaw cycles and tube transfers, and delayed storage are expressed as mean percentage change compared to the values of the reference samples. Normality tests were not productive due to the small sample size hence the statistical significance of observed variations was assessed through non-parametric analysis of variance (ANOVA) in repeated measures (Friedman’s test) followed by post-test Dunn’s multiple comparison tests compared to one control reference value using GraphPad Prism. A *p*-*value* < 0.05 was regarded as statistically significant. However, given the small sample size statistical values may not reach significance therefore a change of >20% from reference values was also considered relevant.

## Results

To investigate the preanalytical variations due to non-specific binding of α-syn species to storage tubes, the effect of different types of polypropylene tubes, use of non-ionic detergents, and multiple tube transfers on measured t- and o- α-syn concentrations was tested. The stability of the α-syn species (t- and o-α-syn) against multiple freeze-thaw cycles and delayed storage was also tested.

## Siliconized Tubes Showed the Least Adsorption for Both Total- and Oligomeric-α-syn Levels

**Figure 1:**
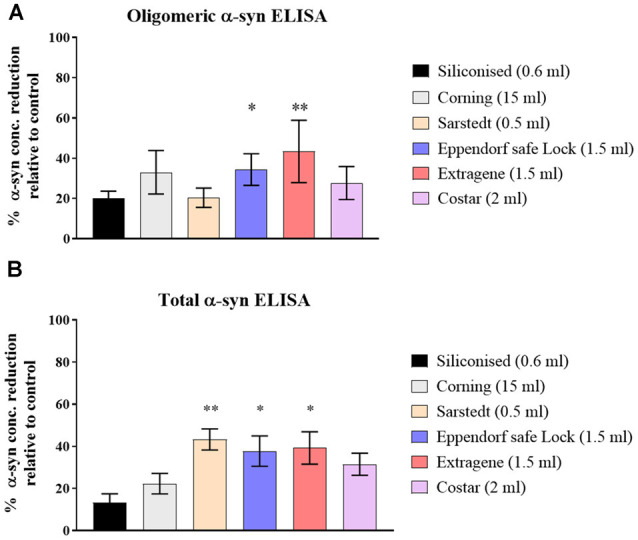
Assessment of α-syn levels in different collection tube types. **(A)** O-α-syn, **(B)** t-α-syn levels in samples collected in different types of polypropylene (PP) tubes relative to control tube type (Nunc-1.8ml). Measurements were taken in duplicates from three cerebrospinal fluid (CSF) samples and results are expressed as the mean percentage reduction in CSF α-syn levels relative to control (Nunc tube). Error bars indicate standard error of the mean (± SEM). **p < 0.01; *p < 0.05.

Three CSF pools were distributed and incubated in six types of polypropylene tubes from different vendors (Table 1) then measured for the t- and o-α-syn concentrations. Results in Figure 1 are expressed as the percentage reduction in t- and o-α-syn levels in the six tube types relative to the control storage tube Nunc tubes (NUNC, Rochester, NY, USA). Siliconized polypropylene tubes showed the least reduction in both o-α-syn (19.5 ± 2.1% reduction) and t-α-syn (13.5 ± 7.8% reduction) concentrations. However, the results showed that the different types of storage tubes affect the levels of the two α-syn species differently. Samples incubated in Sarstedt tubes showed the second-lowest reduction in o-α-syn levels (22.5 ± 0.7%) nearly similar to levels recovered when siliconized polypropylene tubes were used whereas using the same Sarstedt tubes, a significant reduction in t-α-syn levels (44% reduction, *p* = 0.003) was observed. For measurements of both t- and o-α-syn levels, the use of Eppendorf Safe Lock, and Extragene tubes showed a significant reduction in the measured levels by 23 ± 4.2% (*p* = 0.025) and 35 ± 2.8% (*p* = 0.042) for t-α-syn and by 34 ± 13.4% (*p* = 0.049) and 51.5 ± 0.7% (*p* = 0.004) for o-α-syn levels, respectively.

## The Use of Non-ionic Detergent Decreased Adsorption and Improved Sample Recovery

**Figure 2:**
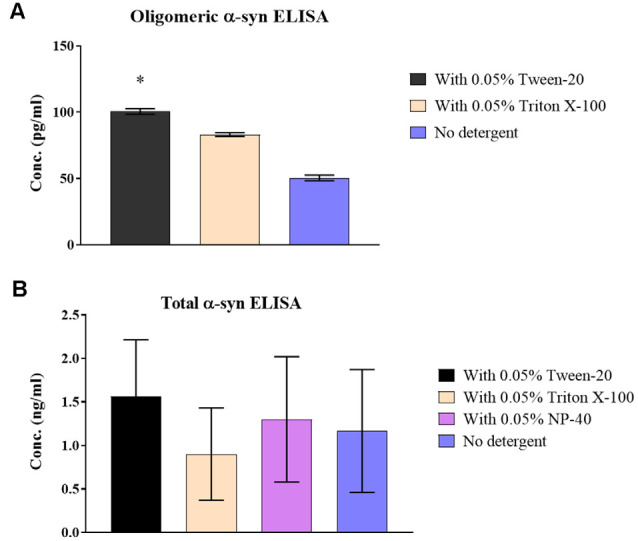
Effect of non-ionic detergents on α-syn levels in samples. **(A)** O-α-syn, **(B)** t-α-syn concentration levels in CSF samples treated with non-ionic detergents Tween-20, Triton X-100, and NP-40 (total α-syn ELISA only) compared to no treatment. Results are expressed as the mean of duplicate measurements (*n* = 3) and error bars indicate the standard error of the mean (±SEM). *p < 0.05.

Treating CSF samples with non-ionic detergent showed a significant increase of 49.2 ± 3.2% in recovered o-α-syn for Tween-20 treated samples (*p* = 0.046) while levels Triton X-100 treated samples increased by 39.2 ± 3.2% although statistical significance was not reached (Figure 2A). T-α-syn levels however showed variability in results with no clear effect of non-ionic detergents on protein recovery (Figure 2B).

## Multiple Tube Transfers Decreased Total-α-syn Levels Measured Due to Adsorption to Tube Walls

**Figure 3:**
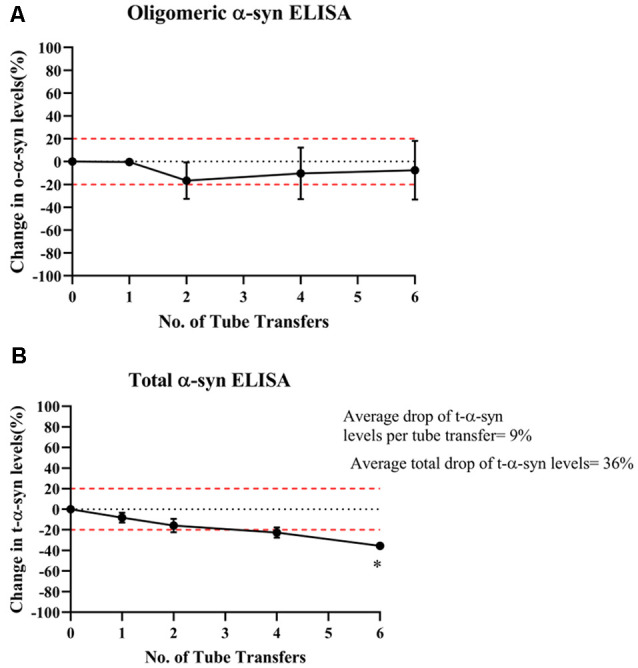
Effect of multiple tube transfers on α-syn levels in samples. **(A)** O-α-syn, **(B)** t-α-syn levels in CSF samples subjected to multiple tube transfers. Measurements were taken in duplicates from three CSF samples and results are expressed as the mean percentage change in CSF α-syn levels. Error bars indicate the standard error of the mean. Red dotted lines represent reference lines at ±20% of the reference value. *p < 0.05.

In contrast, o-α-syn levels remained relatively stable following multiple tube transfers (Figure 3A) whereas t-α-syn steadily decreased by an average of 9% reduction per tube transfer with the statistically significant difference observed after at 6 tube transfers (*p* = 0.018; Figure 3B).

## Multiple Freeze-Thaw Cycles Significantly Decreased Levels of Oligomeric-α-syn Measured in Samples

**Figure 4:**
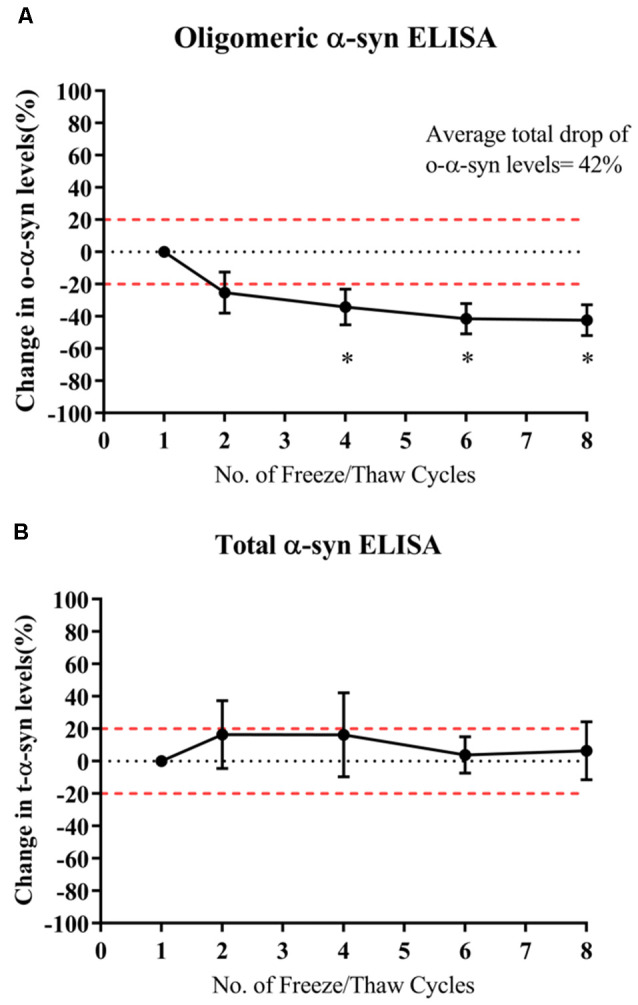
Effect of multiple freeze-thaw cycles on α-syn levels in samples. **(A)** O-α-syn, **(B)** t-α-syn levels in CSF samples subjected to multiple freeze-thaw cycles. Measurements were taken in duplicates from three CSF samples expressed as a mean percentage change in CSF α-syn levels. Error bars indicate the standard error of the mean. Red dotted lines represent reference lines at ±20% of the reference value. *p < 0.05.

O-α-syn levels significantly decreased by 34% after the fourth thaw cycle and continued significantly decreasing with each cycle to a total drop of 42% after seven cycles (*p*=0.021; Figure 4A). However, levels of t-α-syn remained stable against freeze-thaw cycles (Figure 4B).

## Delayed Storage Had Little or No Impact on α-syn Measured in Samples

**Figure 5:**
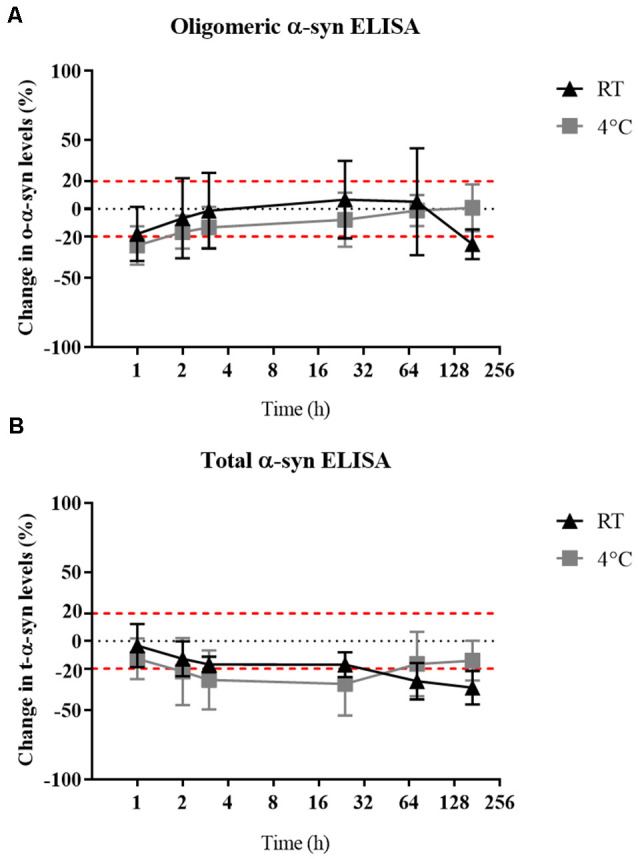
Effect of delayed storage of samples on their α-syn levels.** (A)** O-α-syn, **(B)** t-α-syn levels in CSF samples delayed storage by: 0, 1, 2, 4, 24, 72, or 168 h at 4°C or room temperature (RT). Measurements were taken in duplicates from three CSF samples expressed as the mean percentage change in CSF α-syn levels. Error bars indicate the standard error of the mean. Red dotted lines represent reference lines at ±20% of the reference value.

Oligomeric-α-syn levels remained relatively stable despite a delay in sample storage (Figure 5A). While a gradual decrease in the measured t-α-syn concentration was observed with prolonged delay in storing samples at −80°C by keeping in RT with the decrease exceeding 20% reduction after 24 h however statistical significance was not reached (Figure 5B). α-syn measurements of samples kept at RT compared to those kept at 4°C prior storage at −80°C, showed no significant difference between both conditions, which could be attributed to the high variability in α-syn levels between samples.

## Discussion

The present study investigates preanalytical considerations for CSF t- and o-α-syn measurements, specifically the impact of potential confounding factors that can occur during sample handling, processing, and storage. To the best of our knowledge, similar studies were limited to analyzing the effect of preanalytical confounding factors on t-α-syn measurements but not o-α-syn CSF levels. This is important considering recent studies have shown improved diagnostic accuracy when combining multiple forms of α-syn, mainly the ratios of o- and pS129- α-syn to t-α-syn (Majbour et al., 2016).

First, we explored the non-specific binding or adsorption of α-syn to the storage tube surface. Studies have investigated this effect in Alzheimer’s disease biomarkers, particularly in Aβ_1–42_ peptide which has a hydrophobic C-terminal making it more prone to aggregate and bind non-specifically to the tube surface (Lewczuk et al., 2006; Pica-Mendez et al., 2010; Perret-Liaudet et al., 2012). Similarly, α-syn protein in its aggregated form increases in hydrophobicity thereby increasing its propensity to be lipophilic and “sticky” (Breydo et al., 2012; Lee et al., 2018). As a result, α-syn could also be expected to adsorb to the storage tube surface.

According to recommended standards (Campo et al., 2012; Mollenhauer et al., 2017a) concerning storage tubes, we used Nunc PP tubes as reference tubes to test the effect of different PP tubes based on previously published work (Pica-Mendez et al., 2010). We demonstrated variable differences in CSF α-syn levels measured using PP tubes from different vendors. A similar observation was previously made in a study by Perret-Liaudet et al. (2012) for amyloid beta. They showed that PP tubes from different vendors resulted in different adsorption ratios despite the uniform use of polypropylene. A closer look revealed that the compositions of the tubes differed in that some were made from pure PP while others were co-polymers with other chemicals such as polyethylene (Perret-Liaudet et al., 2012). Our results showed that α-syn adsorption was largely attenuated by the use of siliconized polypropylene tubes, with a significant decrease in adsorption (~20% and ~25%) reduction of adsorption in siliconized tubes compared to other tubes for o- and t- α-syn levels, respectively. Silicone coating provides the tubes with a non-binding surface which significantly reduces adsorption of sticky proteins to the tube walls and low retention of protein in the tube (Chen et al., 2004). A trend of minimal retention of o-α-syn was noted when samples were stored in tubes with a smaller surface-volume ratio. This could be a factor influencing the adhesion of α-syn to storage tube walls as previous studies have reported that surface-volume ratio can potentially influence the measured concentrations of other CSF biomarkers (Vanderstichele et al., 2016; Delaby et al., 2019).

Previous studies showed the advantages of using non-ionic detergents such as Tween-20 in preventing loss of CSF amyloid proteins due to tube adsorption (Mollenhauer et al., 2008, 2017a; Pica-Mendez et al., 2010). In our study, the use of non-ionic detergents, Tween-20 and Triton X-100, was found to mitigate the magnitude of reduction in CSF o-α-syn levels, with Tween-20 detergent providing more effective results than Triton X-100 by at least 10%. Despite showing better stability against the different types of PP tubes, o-α-syn levels were remarkably affected by the absence of non-ionic detergents. It may be worth noting this observation as not solely a mechanism of reduced adsorption of o-α-syn but a possible result of effects of the detergents on the antibody-antigen complexes or formation of oligomers. Results of the non-ionic detergent effect on CSF t-α-syn levels were inconclusive due to the high variability noted among samples. Further experiments with a larger sample set are needed to better conclude the preventive impact provided by the use of detergents on CSF t-α-syn adsorption levels.

Examining the effect of multiple tube transfers, o-α-syn levels remained almost unaffected despite multiple tube transfers, while t-α-syn levels continuously declined with almost 36% of t-α-syn lost after the sixth transfer. Considering the capture and detection antibodies employed in our total ELISA (Syn-140, sheep anti-α-syn polyclonal antibody, and 11D12, mouse anti-α-syn monoclonal antibody) (Majbour et al., 2016), the assay measures monomers, aggregates, phosphorylated S129, and nitrated forms of α-syn. While our oligomeric ELISA, utilizing Syn-O2 (o-α-syn specific monoclonal antibody) for capture, measures aggregates of full-length α-syn. The differences between the impact of multiple tubes on the named analytes could be explained by the difference in the range of forms captured by each ELISA.

Investigating the stability of CSF α-syn over repeated freeze-thaw cycles is essential in dictating the conditions and methods used in storing CSF samples before testing. Results of our study show that t-α-syn levels are not affected by multiple freeze-thaw cycles whereas o-α-syn levels steadily decreased with each freeze-thaw cycle. However, another study demonstrated that t-α-syn levels declined with each cycle to up to 50% reduction after six cycles (Campo et al., 2012). Given the variability of reported results, it would be best to follow practices such as aliquoting CSF that have been recommended to avoid multiple freeze-thaw cycles and to not exceed two freeze-thaw cycles (Campo et al., 2012; Mollenhauer et al., 2017a).

In terms of α-syn stability against delayed sample storage, we found o-α-syn levels to be more stable than t-α-syn which showed a decrease in levels by 20% after 24 h of delayed storage, independent of the temperature the samples were kept during this delay. Previous reports showed a decrease in t-α-syn levels up to 40% when stored at 4°C for 4 days (Campo et al., 2012 ) while another study found no significant difference in α-syn levels in samples when exposed to prolonged delay in storage, 20 min–48 h, at either RT or 4°C after sample collection (Mollenhauer et al., 2017a). Samples frozen at −20°C for 1 month were stable and did not show a difference compared to reference samples directly frozen at −80°C (data not shown). The susceptibility of t-α-syn stability to delayed storage suggests the importance of sample handling guidelines stressing immediate processing and storage of samples.

To the best of our knowledge, this is the first study to assess the stability of o-α-syn along with t-α-syn against preanalytical confounding factors. The study also has some limitations: first, the lack of statistical power due to the small sample sets might have confounded our conclusions. Second, CSF sample pools from a biobank were used for our experiments. This meant that the samples used were already subjected to at least one freeze-thaw cycle and were stored long-term at −20 or −80°C before being exposed to our experimental conditions. Therefore, any alterations in α-syn chemistry that occurred due to this may have affected its stability and could not be assessed. Validation experiments to confirm the long-term stability of α-syn measurements after correct storage would be valuable to include in future studies.

## Conclusion

Between lumbar puncture and laboratory analysis, collected CSF samples undergo variable sampling and storage methods, potentially different between individuals and sites processing the samples, which are often unknown to the scientists running the assays. We have investigated α-syn stability against these preanalytical variables and have shown that α-syn levels measured are affected, to different degrees, by the type of storage tube used, the addition of detergent, number of tube transfers, number of freeze-thaw cycles, and temperature and time to freeze. The impact of preanalytical factors was noticeably different between o- and t-α-syn forms, which means cautious awareness is required when analyzing the different forms. Our study revealed o-α-syn to be seemingly more stable, compared to t-α-syn, against most of the preanalytical variables tested, particularly adsorption to tube surface and delayed storage processing to −80°C. However, the opposite was noted when samples were exposed to multiple freeze-thaw cycles as t-α-syn levels remained stable while o-α-syn levels dropped considerably. In summary, the use of siliconized tubes, non-ionic detergents, immediate storage of samples, minimal freeze-thaw cycles, and tube-transfer provide the best preservation of t- and o-α-syn species. Our results emphasize the value of evaluating the impact of preanalytical factors on other subforms of α-syn emerging as potential biomarkers for synucleinopathies such as phosphorylated or truncated α-syn and employing different assessment methods such as protein misfolding cyclic amplification (PMCA) or Real-time quaking-induced conversion (RT-QuIC). Additionally, application studies assessing the correlation between the stability of α-syn forms and their impact on biomarkers studies would also be of great benefit to the research community.

## Data Availability Statement

The raw data supporting the conclusions of this article will be made available by the authors, without undue reservation.

## Author Contributions

NM and OE-A: study design and supervision. IA, NM and EW: conducting of the experiments. IA and NM: data analysis. IA: writing the first draft of the manuscript and incorporating revisions from other authors. IA, NM, WB, BM, CT and OE-A: data interpretation and critical revision of the manuscript for important intellectual content. OE-A: final review of the draft and approval to submit for publication. All authors contributed to the article and approved the submitted version.

## Conflict of Interest

The authors declare that the research was conducted in the absence of any commercial or financial relationships that could be construed as a potential conflict of interest.
